# Exploratory Study of Associations and Agreement between Prognostic Patient-Registered Factors, Physiotherapists’ Intuitive Synthesis, and Patient-Reported Factors in Whiplash-Associated Disorders

**DOI:** 10.3390/jcm12062330

**Published:** 2023-03-16

**Authors:** Rob A. B. Oostendorp, Gwendolyne G. M. Scholten-Peeters, Jan Mulder, Emiel Van Trijffel, Geert M. Rutten, Margot De Kooning, Marjan Laekeman, Nathalie Roussel, Jo Nijs, J. W. Hans Elvers

**Affiliations:** 1 Scientific Institute for Quality of Healthcare, Radboud University Nijmegen Medical Centre, 6525 Nijmegen, The Netherlands; 2 Department of Manual Therapy, Faculty of Medicine and Pharmacy, Vrije Universiteit Brussel, 1050 Brussels, Belgium; 3 Pain in Motion International Research Group, Faculty of Physical Education and Physiotherapy, Vrije Universiteit Brussel, 1050 Brussels, Belgium; 4 Practice Physiotherapy and Manual Therapy, 5473 Heeswijk-Dinther, The Netherlands; 5 Amsterdam Movement Sciences Program Musculoskeletal Health, Department of Human Movement Sciences, Faculty of Behavioral and Movement Sciences, Vrije Universiteit Amsterdam, 1081 Amsterdam, The Netherlands; 6 Department of Functional Dentition and Prosthetic Dentistry, Radboud University Nijmegen Medical Centre, 6525 Nijmegen, The Netherlands; 7 Department of Public Health and Research, Radboud University Nijmegen Medical Centre, 6525 Nijmegen, The Netherlands; 8 Ziekenhuisgroep Twente, ZGT Academy, 7609 Almelo, The Netherlands; 9 Experimental Anatomy Research Group (EXAN), Department of Physiotherapy, Human Physiology and Anatomy (KIMA), Faculty of Physical Education and Physiotherapy, Vrije Universiteit Brussel, 1050 Brussels, Belgium; 10 Research Program of Organization of Healthcare and Social Services, School of Health Studies, HAN University of Applied Science, 6826 Nijmegen, The Netherlands; 11 Department of Physiotherapy, Human Physiology and Anatomy, Faculty of Physical Education and Physiotherapy, Vrije Universiteit Brussel, 1050 Brussels, Belgium; 12 Department of Physiological Psychology, Otto-Friedrich University of Bamberg, 96047 Bamberg, Germany; 13 Department of Physiotherapy and Rehabilitation Sciences (MOVANT), Faculty of Medicine and Health Sciences, University of Antwerp, 2000 Antwerpen, Belgium; 14 Department of Physical Medicine and Physiotherapy, University Hospital Brussels, 1090 Jette, Belgium; 15 Methodological Health-Skilled Institute, 6641 Beuningen, The Netherlands

**Keywords:** whiplash associated disorders, prognostic factors, prognosis, functional recovery, routinely collected data, clinical reasoning, clinical intuition

## Abstract

Background: A large proportion of people who sustain a whiplash injury will have persistent pain, disability, and participation problems. Several prognostic factors for functional recovery have been reported in the literature but these factors are often evaluated based on differing implementations in clinical practice. Additionally, physiotherapists also rely on their clinical intuition to estimate the functional prognosis of their patients, but this is seldom measured in experimental research. Furthermore, no study to date has explored the associations between clinical intuition, clinically estimated factors, and objectively measured factors for functional recovery of patients with Whiplash-Associated Disorders (WAD). Aim: The aim of this exploratory study is to evaluate associations between prognostic factors for functional recovery, based on routinely collected data in a specialized primary care physiotherapy practice in a consecutive sample of patients (n = 523) with WAD. Methods: Three sources of prognostic factors were selected: (1) physiotherapists’ synthesis of clinical intuition in terms of high-risk, inconclusive risk, or low-risk for functional recovery, (2) patient-registered factors from history taking, and (3) patient-reported prognostic factors derived from questionnaires. Prognostic factors were selected based on the literature, recommendations in Dutch clinical practice guidelines, and consensus between experts. Spearman’s rank correlation coefficients were calculated to explore the associations between sources of prognostic factors, using a cutoff ≥0.25 for acceptable association. Results: Associations between physiotherapists’ intuitive synthesis and patient-registered variables were substantial (*r_s_* = 0.86), between patient-registered variables and patient-reported variables fair (ranging from 0.30 to 0.41) to substantial (ranging from 0.69 to 0.73), and between physiotherapists intuitive synthesis and patient-reported variables fair (ranging from 0.30 to 0.37). Conclusion: When estimating prognosis for functional recovery using clinical reasoning, physiotherapists should integrate patients’ registered experience of their course of recovery, as well as the timeline after an accident, with their own synthesis of clinical intuition regarding prognostic factors in patients with WAD.

## 1. Introduction

Neck pain after whiplash injury is often considered a result of soft tissue lesions [1,2]. Acceptance of this soft-tissue healing model implies that the majority of patients with whiplash-related injury will recover within the expected time frame (6–12 weeks) [3]. Neck pain after whiplash injury that persists beyond the expected recovery time is considered to be posttraumatic chronic neck pain or a chronic whiplash-associated disorder [4].

However, the unitary time dimension of soft-tissue healing after whiplash injury is under discussion [5,6]. The impact of whiplash injury is restricted not only to lesions of the cervical spine but also affects structures of the peripheral and central nervous systems. This can lead to a variety of dysfunctions including impairments of sensory, movement-related, hearing and vestibular functions (dizziness, tinnitus, loss of balance), mental functions (cognitive and sleep functions), as well as activity limitations and participation restrictions. Pain and sensory functions are indicative of sensitization of the peripheral and central nervous systems [7,8,9,10,11]. The high prevalence of all these complaints, combined with the lack of relationship between collision-related factors and signs of tissue injury, are suggestive of a central neurological disorder rather than a peripheral traumatic disorder [5,6]. This category of nociplastic pain is mechanistically distinct from nociceptive pain, which is caused by ongoing inflammation and nociceptive information [12,13,14]. Up to 50% of patients will develop long-term posttraumatic chronic neck pain and experience long-term impairments in bodily functions, activity limitations, and restrictions to participation [9,11,15,16].

Together these data imply that prognostic factors for functional recovery are not only dependent on physiological soft-tissue healing but also on functioning of the central nervous system, such as the motor control system and pain modulating system [17,18,19,20,21,22,23].

Based on the International Classification of Functioning (ICF), a shift in focus has taken place from anatomical structures to actual functioning of the neck region when describing health and health-related states in patients with WAD [24,25]. As part of this conceptual shift from anatomical structure to functioning, physiotherapy assessments in patients with WAD were extended to include prognostic health profiles [26,27]. A prognostic health profile is often defined as “A situation or condition, or a characteristic of a patient, that can be used to estimate the chance of recovery from a disease or the chance of the disease recurring” [28]. Applied to patients with WAD, a prognostic health profile can be seen as a complex interaction between positive (low risk), inconclusive, and negative (high risk) factors which can be used to estimate the probability of functional recovery in patients with WAD, and to decide indicator-based treatment goals and treatment [29,30]. Normally a combination of high-risk, inconclusive-risk and low-risk prognostic factors are reported, profiles in which either low- or high-risk factors may predominate.

Efforts to provide a realistic prognosis for the individual trajectory of recovery in patients with WAD have led to a multitude of studies aiming to identify explanatory prognostic factors [7,8,9,31,32,33,34,35,36,37,38,39,40]. These studies have explored a wide range of prognostic factors (sociodemographic, biomedical, psychological, social) known to be associated with recovery. The most consistent prognostic factors for functional recovery are baseline pain intensity (postinjury pain), functioning (disabilities), catastrophizing, expectations of recovery, coping, and fear avoidance. However, systematic reviews of clinical prediction rules (CPRs) for prognosis of functional recovery in patients with WAD concluded that most CPRs are not yet externally validated nor assessed for clinical applicability. Consequently, CRPs cannot be used to estimate functional recovery in clinical practice [41,42,43,44].

Physiotherapy is one of the most commonly applied treatment options in patients with WAD [45,46,47]. However, many evidentiary gaps remain in terms of diagnostics, prognostics, and treatment, as well as concerning patient-related outcome measurements in patients with acute or chronic WAD. Routinely collected data (RCD) describing real practice populations, such as patients with WAD, can fill these evidentiary gaps and is a recommended supplementation of real-world evidence [48,49,50]. However, exploring and analyzing prognostic factors for functional recovery based on routinely collected data (RCD) is a challenge, as this data include real-world data originating from a variety of sources such as patient records, physiotherapists’ intuitive and implicit clinical reasoning, as well as patient-reported data derived from validated questionnaires.

Implicit clinical reasoning of experienced physiotherapists is normally based on pattern recognition, but when confronted with a complex, unfamiliar health problem the experienced physiotherapist places more reliance on hypothetico-deductive reasoning. Health problems of patients with (chronic) WAD are often complex and multidimensional and require a particular clinical intuition based on a particular pattern recognition. Clinical intuition has been described as an outcome of interactions between a particular clinician or group of clinicians and a particular patient or patient group in a specialized setting [51].

To our knowledge, identification of the prognostics of functional recovery in patients after a whiplash injury, based on RCD in a specialized primary care physiotherapy practice, has received little attention. Because RCD offer unique opportunities for collecting ecologically valid data, this represents an important gap in knowledge.

The definition of prognostic factors for functional recovery based on a synthesis of clinical intuition, on data registered in patient records, and on valid patient-reported measurements of the most consistent prognostic factors for recovery or non-recovery, would be an important addition to clinical physiotherapy practice. At the moment, we lack insight into the way in which prognostic factors in patients with WAD are coherently established in primary care physiotherapy practice.

We report an exploratory study of prognostic factors for functional recovery based on real world data in patients with WAD. This study evaluated the association and agreement between prognostic factors of functional recovery as assessed through physiotherapists’ synthesis of clinical intuition, as well as relevant clinical data from a patient record registry and data on patient-reported variables, comparing these to each other as an indication of the complexity of functional recovery in patients with WAD.

## 2. Materials and Methods

### 2.1. Design

This study was based on an existing dataset (routinely collected dataset whiplash-associated disorders [RCD-WAD]) assembled over a period of 16 years. Details of the design (a longitudinal observational study) and execution of that original prospective cohort study have been published elsewhere [30,52,53].

The Medical Ethics Committee of Radboud University Medical Centre Nijmegen, The Netherlands, waived the requirement for ethical approval. Retrospective research based on anonymized patient files does not fall within the scope of the Medical Research Involving Human Subjects Act because subjects are not physically involved in the research.

### 2.2. Participants

Patients with whiplash-related symptoms were referred by general practitioners or medical specialists to two primary care specialized physiotherapy practices in the Netherlands. Annually, approximately 50 to 60 patients were referred after a whiplash injury. All patients who met the Quebec Task Force Classification of WAD-1, WAD-2 or WAD-3 were assessed [54]. Following initial screening related to the 5Ds +1 (dizziness, diplopia, drop attacks, dysarthria, dysphagia + nausea), patients with ≥2 Ds were referred back to their general practitioner due to probably serious underlying pathology, and thus excluded from the study.

Eight physiotherapists participated in the study, collecting routinely data of referred patients with WAD. All patients were assessed by one of the eight physiotherapists. The participating physiotherapists received updates in accordance with the content of the most recent CPG Whiplash and Physiotherapy and the adapted patient record files, as explained in three meetings of 3 h. The junior physiotherapists (n = 6) were supervised and trained in clinical assessment and intuition by the senior physiotherapists (n = 2). The synthesis of the participating physiotherapists’ clinical intuition is based on special pattern (re-)cognition regarding patients in all phases (acute, subacute, and chronic) after a whiplash-related injury, facilitating recognition and interpretation of communication.

The mean age of the physiotherapists at the beginning of the registration period for routinely collected data was 46.3 years (SD 5.6). The range of experience at that time regarding assessment of patients with WAD varied between 6 and 28 years.

### 2.3. Data Collection

Clinical data on patients with WAD (RCD-WAD) were routinely collected over a period of 16 years (1996–2011) and were used in the context of the project Quality of Physiotherapy and WAD. After cleaning and processing of the dataset, the analysis of the RCD-WAD dataset started in 2016 in the context of the project Quality of Physiotherapy and WAD [55].

The current analysis concerns a selection of sociodemographic characteristics and prognostic factors for functional recovery in patients with WAD under the umbrella of exploratory studies with a prognostic aim (description and association) [56]. All data regarding clinical reasoning were recorded during the 1 h session, with questionnaires scores added during the second session.

### 2.4. Procedure

Prognostic factors for functional recovery in this study were selected based on the literature [32,37,38,39,40,41], recommendations in the Dutch clinical practice guideline (CPG) Physiotherapy and Whiplash/Neck Pain [26,27,57], and expert consensus.

Data were extracted from the existing RCD-WAD dataset comprising a consecutive sample of 523 patients with WAD [52].

### 2.5. Selection of Variables of Prognostic Factors for Functional Recovery

**Figure 1:**
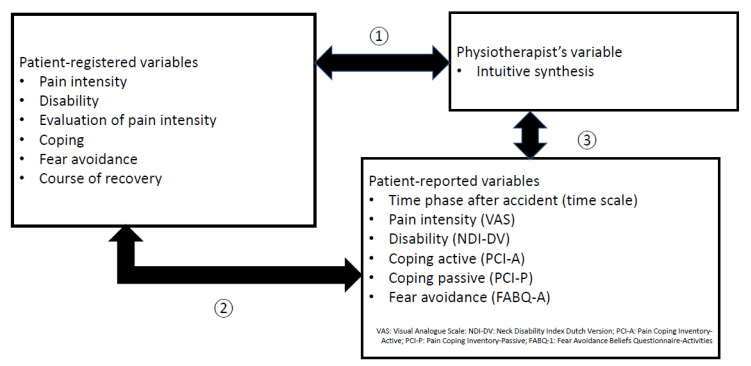
Three steps of explorative study of associations of prognostic factors for functional recovery of patients with Whiplash-Associated Disorders (WAD).

A three-step scheme for the exploratory study of associations between the selected variables of the prognostic profile is presented in Figure 1.

The first selection of prognostic factors was based on physiotherapists’ clinical judgment (including past experience, pattern recognition, and intuition) as an overall synthesis of variable “low risk”, “inconclusive risk”, or “high risk” for functional recovery. Physiotherapists recorded their clinically intuitive synthesis as the first item in the patient’s registration form.

The second selection of prognostic factors was composed on the basis of registration of relevant prognostic variables in patient records: pain intensity (mild, moderate, severe), functioning/disability (mild, moderate, severe), evaluation of pain (decreasing, inconclusive, increasing), coping (active, inconclusive, passive), fear avoidance (no, inconclusive, yes), and patients’ experience of the course of recovery (normal, no change, delayed).

The third selection of prognostic factors was based on patient-reported measurements of time scale after accident (0–3 weeks, 4–12 weeks, 3 ≥ 6 months), questionnaires on pain intensity (Visual Analogue Scale [VAS]), disability (Neck Disability Index [NDI]), coping (Pain Coping Inventory [PCI]), and fear avoidance (Fear Avoidance Beliefs Questionnaire [FABQ]).

Clinimetric properties of the four questionnaires have shown good reliability and validity [58,59,60,61,62].

Pain intensity was measured using the VAS for pain, which consists of a horizontal 100 mm line scored from 0 (no pain) to 100 (worst imaginable pain). Pain intensity is classified in three classes: mild (0–30 mm mild), moderate (31–60 mm), and severe (61–100 mm) [63].

Functioning or disability was measured using the NDI Dutch Version (NDI-DV). The NDI is a self-report questionnaire that measures activity limitations due to neck pain resulting from whiplash-related injuries. The NDI consists of ten items that address pain intensity, personal care, lifting, reading, headache, concentration, work, driving, sleeping, and recreation. Each item is scored from 0 (no activity limitation) to 5 (major activity limitation). The total score range is 0–50, with increasing scores representing increasing impairments and disabilities due to neck pain. The total NDI score is categorized as three classes: mild (0–14 points), moderate (15–24 points), and severe (25–50 points) [64].

The Pain Coping Inventory (PCI) is a 33-item questionnaire measuring active coping (PCI-Active: 12 items; total score range 12–48 points) and passive coping (PCI-Passive: 21 items; total score range 21–84 points). Items are scored on a 4-point Likert scale ranging from 1 (hardly ever) to 4 (very often) depicting frequency with which active and passive strategies are applied when dealing with pain. The total PCI-A score is assigned as one of three classes of active coping: low risk (36–48 points), inconclusive risk (24–35 points), and high risk (12–23 points). Similarly, the total score on PCI-P is categorized into three classes of passive coping: low risk (21–42 points)’, inconclusive risk (43–63 points), and high risk (64–84 points) [65].

The FABQ-Dutch Version (FABQ-DV) is a 16-item questionnaire measuring fear avoidance beliefs regarding physical activities (FABQ-DV-A: 4 items; total score range: 0–24 points) and work-related activities (FABQ-DV-W). FABQ-DV-A is only used for the classification of the prognostic health profile for functional recovery. Items are scored on a 7-point Likert scale ranging from 0 (completely disagree) to 6 (completely agree). As above, the total score of FABQ-DV-A is categorized as one of three classes of fear avoidance: low risk (0–10 points), inconclusive risk (11–15 points), and high risk (16–24 points) [66].

### 2.6. Statistical Analysis

Descriptive statistics were used to characterize the study population’s demographics, including a selection of prognostic factors related to accident- and health-related characteristics. Percentage scores were calculated for selected ordinal variables or dichotomized variables.

Spearman’s rank correlation coefficients (*r_s_*) were calculated to explore associations between the selected variables of the three types of prognostic factors for functional recovery (physiotherapists’ synthesis of clinical intuition, variables based on patients’ records, and patients’ scores on questionnaires). The following criteria were used to indicate the strength of associations: 0.00 to 0.25: weak association; 0.25 to 0.50: fair association; 0.50 to 0.70: moderate association; 0.70 to 0.90: substantial association; ≥0.90: perfect association [67]. Correlation coefficients of ≥0.25 were considered cutoff points for acceptable associations [67]. For all associations, a *p* value ≤0.05 was considered statistically significant.

For each pair of variables, 3 × 3 tables were constructed. Percentages of agreement were calculated for the pair of variables with a correlation coefficient ≥0.25. The software program Statistix 10 was used for statistical analysis [68].

## 3. Results

### 3.1. Participants

A consecutive sample of 529 patients was originally included. Six patients were referred back to their general practitioner due to suspicion of serious pathology. Selected sociodemographic characteristics (n = 523) are presented in Table 1. More than 40% of patients (n = 232; 44.4%) visited the practice following referral by a general practitioner or self-referral in the period 4–12 weeks after an accident. Most patients (n = 406; 77.6%) were classified as WAD-2. A complete overview of the baseline characteristics of participating patients with WAD has been published elsewhere [52].

### 3.2. Scores of Prognostic Factors for Functional Recovery

The scores of the selected prognostic factors are presented in Table 1.

The physiotherapists’ intuitive synthesis for functional recovery led to the classifications “low risk” in 43 patients (8.2%), “inconclusive risk” in 329 patients (62.9%), and “high risk” in 151 patients (28.9%).

Most prognostic patient-registered factors were “inconclusive” or “high risk” for functional recovery after accident. Pain intensity was “moderate” in 407 patients (77.8%) and activity limitation “severe” in 290 patients (55.4%). Increasing pain since the accident (n = 328; 62.7%), passive coping (n = 307; 58.7%), and fear avoidance (n = 303; 57.9%) were potential high-risk factors for functional recovery. Patients’ experience of the course of recovery since whiplash-related accident was either “no change” (n = 339; 64.8%) or “delayed” (n = 144; 27.5%).

Patient-reported variables revealed that pain intensity on VAS was moderate in 266 patients (50.9%) and severe in 242 patients (24.2%). Activity limitations on NDI-DV were severe in 406 patients (77.6%). Coping on PCI-A was inconclusive or high risk for active coping in 337 patients (64.4%) and 127 patients (24.3%), respectively, and inconclusive or high risk for passive coping in 263 patients (50.3%) and 153 patients (29.3%), respectively. Fear avoidance was scored as high risk in 346 patients (66.2%).

### 3.3. Associations between Selected Prognostic Factors

Correlation coefficients between the selected variables are presented as a correlation matrix in Table 2. The total number of correlation coefficients was 48; Step 1: n = 6 (<0.25: n = 5; ≥0.25: n = 1); Step 2: n = 36 (<0.25: n = 29; ≥0.25: n = 7); and Step 3: n = 6 (<0.25: n = 2; ≥0.25: n = 4).

Correlation coefficients between (step 1) physiotherapists’ synthesis of clinical intuition and patient-registered factors (patients’ experience of course of recovery) was substantial (*r_s_ =* 0. 86), and between (step 2) patient-registered factors and patient-reported variables fair to substantial (ranging from 0.30 to 0.73). Coefficients between (step 3) physiotherapists’ intuitive synthesis and patient-reported variables were fair (ranging from 0.30 to 0.37).

### 3.4. Steps of Association and Agreement between Selected Prognostic Factors for Functional Recovery

#### 3.4.1. Step 1

Agreement between physiotherapists’ intuitive synthesis and patients’ experience of the course of recovery after an accident was 91.5%.

Based on a 3 × 3 cross table, agreement and disagreement between physiotherapists’ synthesis of low risk for functional recovery and patients’ experience of the normal course of recovery was 7.6% and 0.6%, respectively; between physiotherapists’ synthesis of inconclusive risk and patients’ experience of no change in the course of recovery 59.5% and 3.3%, respectively; and between physiotherapists’ synthesis of high risk and patients’ experience of a delayed course of recovery 24.3% and 4.6%, respectively.

The value of the correlation coefficient between the intuitive synthesis of the physiotherapist and the course of recovery was clustered in a linear relationship and confirmed by the percentages of agreement [67].

#### 3.4.2. Step 2

Fair correlation coefficients were found between the patient-registered variable “patients’ experience of course of recovery” and patient-reported variables with the validated instruments “pain intensity”, “disability”, and “fear avoidance”, ranging from 0.41 to 0.30 (Table 2).

Ratios of the percentages of agreement between the pairs of variables “patients’ experience of course of recovery” and patient-reported variables ranged from 39.9% to 63.6%, illustrating the various degrees of linear correlation for each pair of variables (Table 3). One specific cell in a 3 × 3 table represents the discrepancy between fair correlation coefficients and relatively high percentages of agreement. For instance, the cell between “no change” of course of recovery and moderate pain intensity (n = 213; 40.7%) or inconclusive active coping (n = 255; 48.8%). The percentages of disagreement were the highest between “no change for functional recovery” and severity of pain intensity (n = 122; 23.3%), severity of disability (n = 262; 50.1%) and high-risk of fear avoidance (n = 212; 40.5%).

Correlation coefficients for the patient-reported variable “time phase after accident” and the patient-registered variables “disability” (*r_s_* = 0.73; 59.7%), “coping” (*r_s_* = 0.73; 55.1%), and “fear avoidance” (*r_s_* = 0.69; 54.1%) were substantial (Table 2).

The percentages of agreement between time phase since accident and disability (n = 323; 59.7%), coping (n = 288; 55.1%), and fear avoidance (n = 283; 54.1%) were clustered in a relatively linear relationship for each pair of variables. (Table 4). The percentages of disagreement were highest between “time phase 4–12 weeks” and severity of disability (n = 114; 21.8%), passive coping (n = 128; 24.5%), and fear avoidance (n = 124; 23.7%).

#### 3.4.3. Step 3

Correlation coefficients between the physiotherapist’s intuitive synthesis of risk for functional recovery and the patient-reported factors “pain intensity” (*r_s_* = 0.37; 61.5%), “disability” (*r_s_* = 0.30; 40.5%), “active coping “(*r_s_* = 0.32; 48.8%), and “fear avoidance” (*r_s_* = 0.31; 47.4%) were fair (Table 5).

The percentages of agreement between physiotherapists’ intuitive synthesis of risk for functional recovery and pain intensity (n = 322; 61.5%), disability (n = 212; 40.5%), active coping (n = 255; 48.8%), and fear avoidance (n = 248; 47.4%)) were clustered in a relatively linear relationship for each pair of variables (Table 5).

The percentages of disagreement were highest between physiotherapist’s intuitive synthesis of inconclusive risk for functional recovery and pain intensity (n = 123; 23.5%), severity of disability (n = 256; 48.9%), and fear avoidance (n = 212; 40.5%).

## 4. Discussion

The main findings of our study are that (1) physiotherapists’ synthesis of clinical intuition for functional recovery was substantially associated with the “patients’ experience of the course of recovery since the accident”; (2) “course of recovery” was fairly associated with patient-reported scores on intensities of pain, disability, coping, and fear avoidance; (3) “time phase since accident” was moderately to substantially associated with disability, coping, and fear avoidance registered in patients’ records; and (4) physiotherapists’ intuitive synthesis was fairly associated with patient-reported scores of intensities of pain, disability, coping, and fear avoidance.

### 4.1. Selection of Prognostic Factors for Functional Recovery

A broad range of prognostic factors has previously been registered and measured in the RCD-WAD dataset [52]. We selected those prognostic factors most consistent with the findings of systematic reviews and clinical practice guidelines, also taking the modifiability of the prognostic indicator into account [7,8,9,26,27,57,69]. Physiotherapists’ intuitive synthesis for risk of functional recovery was added as a prognostic factor. Clinical intuition is an unusual prognostic factor in general, particularly in prognostics of patients with WAD.

In some cases, clinical intuition has been found to improve clinical decision-making in psychology, nursing, and family medicine [70,71]. It should be acknowledged that little is currently known about the validity of clinical intuition for risk of functional recovery in patients with WAD. The exploration described here of the associations between physiotherapists’ intuitive synthesis, in terms of low risk, inconclusive risk, and high risk for functional recovery, and the prognostic factors “patients’ experience of course of recovery since the accident”, in terms of normal, no change or delayed, represents a first step in the revaluation of clinical intuition in clinical decision making [72]. It seems likely that participating physiotherapists arrive at their prognosis of functional recovery based on the time phase after accident together with an integrated clinical estimate of intensity of pain, disability, coping, and fear avoidance.

Although the studied data were collected within the framework of the ICF as applied by the Royal Dutch Society for Physical Therapy, future studies should place more emphasis on the assessment of additional social factors when examining prognostic factors for recovery after a whiplash injury. The present work included key biological and psychological factors, but was limited regarding the social dimension of WAD (i.e., only educational level and employment status were assessed). Within the biopsychosocial model, a more comprehensive assessment of potentially relevant social factors seems warranted [73]. For instance, social support potentially affects recovery rate following whiplash injury, as stronger social support is associated with better long-term functioning following whiplash injury [10].

### 4.2. Significance of Correlation Coefficients

Statistical significance of correlation coefficients is very sensitive to sample size. With larger samples (such as n = 523 in this analysis), even values of *r*_s_ < 0.25 can be statistically significant. The interpretation of the absolute size of *p* values needs to take into consideration whether the available sample size was appropriate, given that its value will be smaller in large samples [67]. In this explorative analysis of prognostic factors, Spearman’s rank correlation coefficients of ≥0.25 were considered the cutoff point for an acceptable association. Although many authors report *p* values associated with low correlation coefficients, the strength of the association is more important than the level of significance [67].

The numerous low values of correlation coefficients in the correlation matrix reflect the fact that the physiotherapists’ intuitive synthesis was only weakly associated with prognostic factors in patient records. Most registered prognostic factors were also weakly associated with the prognostic factors reported by the patient via validated questionnaires. Based on a definition of clinical reasoning as a process in which the physiotherapist, interacting with the patient, structures treatment plans and evaluations, associations between clinical variables in the clinical reasoning process are considered one of the most transparent cornerstones in the process of understanding low, inconclusive, and high-risk prognostic factors in relation to the extent of functional recovery in the individual patient [74]. However, clinical practice indicates that physiotherapist’s clinical reasoning and decision making is often not explicit but is frequently based on implicit clinical reasoning, past experience, and pattern recognition. Together, these factors may lead to practice variation between physiotherapists but also within the same physiotherapy practice [75]. Important discussions are needed concerning the challenges faced when seeking to change or sustain the clinical reasoning of physiotherapists [76]. The risk of delayed or incomplete functional recovery from whiplash is a complex construct in clinical reasoning that is not easily captured in an algorithmic model of clinical prediction rules (CPRs) [41,42,73]. This is likely because functional recovery is a multidimensional construct with often misunderstood barriers, including both personal and social characteristics, as well as aspects of the therapeutic relationship [73].

### 4.3. Correlation Coefficients and Percentages of Agreement

The high correlation coefficient between the intuitive synthesis of the physiotherapist and the patient’s course of recovery illustrates the high percentage of agreement, providing insight into the linear association of the two variables. Lower correlation coefficients (e.g., between physiotherapist’s intuitive synthesis and patient-reported measurements of pain intensity, disability, active coping, and fear avoidance) have lower percentages of agreement. The patterns of the associations in relation to high, medium, or low percentages of agreement were more or less linear. The advantage of presenting percentages of agreement is that it allows assessment of the degree of linear association between the two variables [67].

The high degree of agreement between the physiotherapist’s intuitive determination of a risk classification and the degree of recovery after a whiplash-related accident is remarkable. Determining the degree of recovery after a whiplash injury is a process that involves the weighting of both low and high-risk prognostic factors during narrative history taking. The cognition underlying this synthetic process of multiple cues is probably the reason that physiotherapists consider low and high-risk factors in relation to recovery in a reflective process. They arrive at a synthesis on the basis of “inconclusive risk indicators” and evaluations over the course of time, subsequently moving toward a lower or higher risk prognostic indicator for functional recovery, in consultation with the patient.

It is striking that the percentages of disagreement between clinically registered and patient-measured pain severity, disability, coping, and fear avoidance are high, ranging from 20 to 50%, with the course of recovery and the timeline after the accident illustrating the various degrees of correlation between the factors. Patients estimated the course of recovery less favorably and felt the severity of their complaints to be higher in the timeline after the accident compared to physiotherapists. An emotional approach to complaints after a whiplash-related accident is likely more prevalent among patients compared to the cognitive approach taken by the physiotherapist. Physiotherapists tend to give an intuitive risk profile the benefit of the doubt, not knowing whether their assessment for a more optimistic (low-risk) or pessimistic (high-risk) profile is preferable. However, it is likely that they will opt for an optimistic profile, in contrast to the expected recovery as outlined in CPGs.

The next step is to develop a prediction model of associated prognostic factors for functional recovery and time-based treatment assessment.

### 4.4. Modifiability of Prognostic Factors

Systematic reviews generally include numerous prognostic factors without making a distinction regarding modifiability by physiotherapy intervention [69]. At this moment, it is still unclear which factors are directly or indirectly modifiable by physiotherapy intervention. Based on the associations of the prognostic factors discussed here, physiotherapists seem to tailor an individual treatment plan, in terms of goals and interventions, to the patient’s needs. The basic elements of this treatment plan are: (1) time phase after accident, which is markedly associated with disability, coping, and fear avoidance, and (2) the patient’s course of recovery since the accident, which is moderately associated with pain intensity, disability, coping, and fear avoidance. A dynamic plan of treatment, based on the modifiability of a number of prognostic psychological and physical factors by physiotherapy intervention, was developed in 2003 [77] and later adapted in 2018 [30]. Further research is needed to link the modifiable prognostic factors and underlying mechanisms to treatment goals, treatment, and patient-related outcomes.

### 4.5. Clinical Implications

Based on clinical intuition, participating physiotherapists classified the risk of functional recovery as “inconclusive” in about 60% of cases. Like all clinical decision-making, clinical intuition regarding prognosis for functional recovery is an inexact science, as positive, negative, and inconclusive prognostic factors are all typically found in most patients with (chronic) WAD. Uncertainty concerning prognostic factors is not unique to patients with (chronic) WAD. It also applies to patients with (chronic) musculoskeletal pain [78,79].

History taking is an essential competency of every physiotherapist and should be taught in the course of their education. History taking is the basis for gaining insight into the prognostics of a patient’s health problem, including patients with WAD. More specifically, history taking in patients with WAD should always precede physical examination and additional tests.

Each physiotherapist has a personal perception of health-related problems in patients with WAD and will likely make distinct clinical decisions in relation to prognostic factors for functional recovery. Even in the case of the same patient, they may not perceive the same issues. When listening to one history, they may not hear the same history. Physiotherapists often use an implicit conceptual framework that combines anatomical, medical, biomechanical, physiological, psychological, and social frameworks synthesized in a so-called physiotherapeutic conceptual framework [80].

A long-standing gulf separates science from clinical intuition, an issue also found in physiotherapy. Intuition is defined as “a natural ability or power that makes it possible to know something without any proof of evidence” [72]. Intuition is often felt to be inferior compared to “hard” science. Intuition can mislead and may result in inconsistency and bias. It is valid to ask “Why do physiotherapists often arrive at a different assessment of the risk of recovery in patients with WAD compared to the expected assessment on the basis of the CPR WAD algorithm? Why don’t they (simply) follow the CPR-WAD algorithm?” [81]. This disagreement is not unique and discrepancies between objective and subjective measures have been reported for patients with various health problems, including chronic fatigue syndrome [82], low back pain [78,80,81], or WAD [81]. In the latter case, the answer may lie in the many problems facing evidence-based physiotherapy for patients with WAD [45,46,47]. Evidence-based physiotherapy (as currently expressed in clinical practice guidelines [82]) appears to assign excessive weight to external evidence in patients with WAD, which is often accompanied by a neglect of the basic science establishing the biological plausibility of physiotherapy. In our opinion, the time has arrived to re-evaluate the clinical intuition of physiotherapists in the assessment of patients with WAD.

Based on the results of this explorative analysis, we can conclude that intuitive synthesis of clinical intuition, the time line after a whiplash incident, as well as the course of functional recovery over time, are all associated with clinically registered and patient-reported pain intensity, disability, coping, and fear avoidance. This finding has clear implications for an individualized treatment plan and patient-related outcomes. We recommend semi-structured history taking in order to avoid restricting communication between the patient and the physiotherapist, allowing them to jointly confirm the moment of whiplash-related accident and make an inventory of the course of recovery in relation to pain, functioning, coping, fear avoidance, and expectations [83] for recovery as modifiable prognostic factors.

### 4.6. Strengths and Limitations

#### 4.6.1. Strengths

This is the first study to evaluate physiotherapists’ intuitive synthesis on functional recovery in patients with WAD. The RCD-WAD data used originally focused on the quality of physiotherapy care and on the impact of (change in) process and outcome quality factors [30,52,53]. The exploration of the associations and agreements of prognostic factors of functional recovery, especially when based on real-world data, is a new step in understanding the implicit clinical reasoning process of physiotherapists’ clinical decision making.

Studies of associations based on real-world data are required in circumstances where it is unclear which variables are potentially important to the prognosis of functional recovery, especially in the case of a special population such as people with WAD. Association studies can identify candidate prognostic factors, including prognostic factors for functional recovery in patients with WAD. Statistical models in prognostic studies of description and association often involve the use of simple univariate analysis in frequency, proportions, and means, as well as measures of variability (ranges, standard deviation) and the use of bivariate analysis. There are conceptual misunderstandings about the use of multivariable analysis from the perspective of association studies designed to identify candidate prognostic factors. Based on the use of the appropriate steps (description and association) and the right choice of statistical model (univariable and bivariable analysis) in the conceptual framework of prognostic studies [56], we identified candidate prognostic factors for functional recovery in patients with WAD such as physiotherapists’ intuitive synthesis of functional recovery and the patients’ experience of recovery since whiplash-related accident regarding pain intensity, disability, coping, and fear avoidance. The identification of these prognostic factors in the multidimensional and complex construct of functional recovery in patients with WAD is perhaps a next step in the revaluation of particular clinical intuition, pattern recognition and the past experience of physiotherapists in the process of clinical reasoning in patients with WAD.

#### 4.6.2. Limitations

The principal limitation of this explorative study was that two primary care physiotherapy practices in the Netherlands involving eight physiotherapists participated in the collection of clinical data. The generalizability of this explorative study might, therefore, be limited. Nevertheless, although national in scope, the lessons learned from this study will surely resonate with a wider international audience.

Data on patients with WAD were collected in the form of a pen and paper patient record that guided the steps of the clinical reasoning process of physiotherapy care. This pen and paper record was based on the first Dutch CPG Physiotherapy Documentation [83]. All clinical variables were later coded in a statistical program as RCD-WAD. The pen and paper record was not available to other physiotherapy practices, and the data file was only visible to the participating physiotherapists. The accessibility and use of this dataset were restricted to quality improvement of physiotherapy in patients with WAD in participating physiotherapy primary care practices.

## 5. Conclusions

The associations between physiotherapists’ intuitive synthesis of risk for functional recovery, patients’ experiences of recovery after accident, and patient-reported measures of pain intensity, disability, active coping, and fear avoidance were weaker than expected. However, understanding weaker associations is just as important as substantial or fair associations when seeking to comprehend the complexity of functional recovery in patients with WAD. The main finding is nevertheless that physiotherapists’ intuitive synthesis is substantially associated with patients’ experiences of time-related functional recovery.

Physiotherapists in our study frequently classified prognostic factors as “inconclusive risk for recovery”. This classification is probably characteristic of the heterogeneity of our consecutive patient sample and may be explained by the high frequency of subacute and chronic patients and, additionally, may also express physiotherapists’ uncertainty concern functional recovery in patients with WAD. Allowing physiotherapists, a degree of uncertainty when assessing prognostic risk of functional recovery may help improve the process of clinical reasoning.

In addition, further research is needed (1) to better assess physiotherapists’ clinical intuition regarding the complexity of functional recovery in patients with WAD, and (2) to connect modifiable prognostic factors for risk of recovery to treatment goals, treatment, and patient-related outcomes.

## Figures and Tables

**Table 1:** Sociodemographic characteristics and selection of prognostic variables in patients with Whiplash-Associated Disorders.

Total n = 523: n (%) unless Otherwise Stated	
*Sociodemographic characteristics*	
Age in years (mean; SD) − Female − Male	43.4 (13.1) 39.8 (13.4)
Gender (female)	396 (75.7)
Educational level − Lower (primary school) − Intermediate (secondary school) − Higher (post-secondary school)	283 (54.1) 147 (28.1) 93 (17.8)
Employment status − Unemployed/job seeking − On work − Retired	180 (34.4) 277 (53.0) 66 (16.6)
*Classification WAD* *^●^*	
WAD − WAD-1 − WAD-2 − WAD-3 − WAD-4	38 (7.3) 406 (77.6) 79 (15.1) -
*Physiotherapist’s intuitive synthesis*	
Risk of functional recovery − Low risk − Inconclusive risk − High risk	43 (8.2) 329 (62.9) 151 (28.9) *151 (28.9)*
*Patient-registered variables*	
Pain intensity − Mild − Moderate − Severe	33 (6.3) 407 (77.8) 83 (15.9)
Activity limitation/Disability − Mild − Moderate − Severe	24 (4.6) 209 (40.0) 290 (55.4)
Evaluation of pain intensity since accident − Decreasing − Stable − Increasing	11 (2.1) 184 (35.2) 328 (62.7)
Coping − Active − Inconclusive − Passive	211 (40.3) 5 (1.0) 307 (58.7)
Fear avoidance − No − Inconclusive − Yes	220 (42.1) - 303 (57.9)
Patient’s experience of course of recovery since accident − Normal − No change − Delayed	40 (7.6) 339 (64.8) 144 (27.5)
*Patient-reported variables*	
Period since accident − 0–3 weeks − 4–12 weeks − 3 ≥ 6 months	112 (21.4) 232 (44.4) 179 (34.2)
Pain intensity VAS-P^●●^ (score 0-100) − Mild (score 0–30) − Moderate (score 31–60) − Severe (score 61–100)	15 (2.9) 266 (50.9) 242 (24.2))
Activity limitations NDI^●●●^ (score 0–50) − Mild (score 0–14) − Moderate (score 15–24) − Severe (score 25–50)	9 (1.7) 108 (20.7) 406 (77.6)
Coping Pain Coping Inventory^●●●●^ PCI-Active coping (score: 12–48) − Low risk (score 36–48) − Inconclusive risk (score 24–35) − High risk (score 12–23)	59 (11.3) 337 (64.4) 127 (24.3)
Coping Pain Coping Inventory^●●●●^ PCI-Passive coping (score 21–84) − Low risk (score 21–42) − Inconclusive risk (score 43–63) − High risk (64–84)	107 (20.5) 263 (50.3) 153 (29.3)
Fear avoidance Fear Avoidance Beliefs Questionnaire^●●●●●^ FABQ-Activities (score 0–24) − Low risk (score 0–10) − Inconclusive risk (score 11–15) − High risk (score 16–24)	56 (10.7) 121 (21.3) 346 (66.2)

Legend: ^●^ Classification WAD: Whiplash-Associated Disorders: WAD 0: no neck symptoms, no physical sign(s); WAD 1: neck pain, stiffness, or tenderness only, no physical sign(s); WAD 2: neck symptoms and musculoskeletal sign(s); WAD 3: neck symptoms and neurological sign(s); WAD 4: neck symptoms and fracture or dislocation. ^●●^ VAS. Visual Analogue Scale for pain. Horizontal line of 100 mm scored from 0 (no pain) to 100 (worst imaginable pain). ^●●●^ NDI Neck Disability Index 10 items scored from 0 (no activity limitation) to 50 (major activity limitation) points ^●●●●^ Pain Coping Inventory (PCI): 33-item questionnaire measuring active coping (PCI-Active: 12 items (12–48); and passive coping (PCI-P: 21 items (21–81); Items are scored on a 4-point Likert scale ranging from 1 (hardly ever) to 4 (very often). ^●●●●●^ FABQ Fear Avoidance Beliefs Questionnaire: 16-item questionnaire measuring fear avoidance regarding physical activities (FABQ-Active: 4 items (0-24); Items are scored on a 7-point Likert scale ranging from 0 (completely disagree) to 6 (completely agree).

**Table 2:** Steps of exploratory study. Step 1: Correlation matrix between physiotherapist’s intuitive synthesis and patient-registered variables; Step 2: Correlation matrix between patient-registered variables and patient-reported variables; Step 3: Correlation matrix between physiotherapist’s intuitive synthesis and patient-reported variables. Correlation expressed in Spearman Rank Correlation Coefficients (n = 523).

Step 1		Step 2							
Intuitive Synthesis		Reported Variables		TIME Phase since Accident	Pain Intensity VAS-P	Disability NDI	Active Coping PCI-A	Passive Coping PCI-P	Fear Avoidance FABQ-A
	Reg. Variables		Registered Variables						
0.00		Pain intensity		−0.04	0.04	–0.00	0.05	–0.03	–0.02
0.00		Disability		0.73 *	0.00	0.05	–0.04	–0.02	0.03
0.02		Evaluation of pain		–0.05	–0.03	–0.05	0.05	–0.22 *	0.01
0.05		Coping		0.73 *	0.05	0.13 *	–0.05	–0.14	0.10
0.07		Fear avoidance		0.69 *	0.07	0.13 *	–0.04	–0.11	0.10
0.86 *		Course of recovery		0.00	0.41 *	0.30 *	–0.37 *	0.14 *	0.35 *
**Step 3**		Reported Variables		0.02	0.37 *	0.30 *	–0.32 *	0.12	0.31 *
			Intuitive synthesis						

Notes: Rs: Spearman rank correlation coefficients of ≥0.25 as cutoff point; * *p* ≤ 0.05; VAS: Visual Analogue Scale for pain; NDI: Neck Disability Index; PCI-A: Pain Coping Inventory Active; PCI-P: Pain Coping Inventory-Passive; FABQ-A: Fear Avoidance Belief Questionnaire-Activities.

**Table 3:** Step 2. Agreement in 3 x 3 tables between the variables patient’s experience “course of recovery” and patient-reported variables “pain intensity”, “disability”, “coping”, and “fear avoidance” (n = 523).

	Reported Variables	Pain Intensity (VAS) n = 523			Disability (NDI-DV) n = 523			Active Coping (PCI-A) n = 523			Fear Avoidance (FABQ-A) n = 523		
Course of Recovery		Mild n (%)	Moderate n (%)	Severe n (%)	Mild n (%)	Moderate n (%)	Severe n (%)	Low Risk n (%)	Incon. Risk n (%)	High Risk n (%)	Low risk n (%)	Incon. Risk n (%)	High Risk n (%)
Normal n = 40 (7.6%)		**6** **(1.1)**	*28* *(5.4)*	*6* *(1.5)*	**3** **(0.6)**	*25* *(4.8)*	*12* *(2.3)*	**4** **(0.8)**	*21* *(4.0)*	*15* *(2.9)*	**25** **(4.8)**	*6* *(1.1)*	*9* *(1.7)*
Inconclusive n = 339 (64.8%)		*4* *(0.8)*	**213** **(40.7)**	*122* *(23.3)*	*3* *(0.6)*	**74** **(14.1)**	*262* *(50.1)*	*49* *(9.4)*	**255** **(48.8)**	*35* *(6.7)*	*22* *(4.2)*	**105** **(20.1)**	*212* *(40.5)*
Delayed n = 144 (27.5%)		*5* *(1.0)*	*25* *(4.8)*	**114** **(21.8)**	*3* *(0.6)*	*9* *(1.7)*	**132** **(25.2)**	*74* *(14.1)*	*61* *(11.7)*	**9** **(1.7)**	*9* *(1.7)*	*10* *(1.9)*	**125** **(23.9)**
Total agreement		**n = 363** **(63.6)**			**n = 209** **(39.9)**			**n = 268** **(51.3)**			**n = 255** **(48.8)**		

Legend: Incon. risk: inconclusive risk; VAS: Visual Analogue Scale for pain (scores: see Table 1); NDI-DV: Neck Disability Index Dutch Version (scores: see Table 1); PCI-A: Pain Coping Inventory Active (scores: see Table 1); FABQ-A: Fear Avoidance Beliefs Questionnaire Activities (scores: see Table 1).

**Table 4:** Step 2: Agreement in 3 × 3 tables between the variables “time phase since accident” and patient-registered variables “disability”, “coping”, and “fear avoidance” (n = 523).

	Registered variables	Disability n = 523			Coping n = 523			Fear Avoidance n = 523		
Time Phase Since Accident		Mild n (%)	Moderate n (%)	Severe n (%)	Active n (%)	Inconclusive n (%)	Passive n (%)	No n (%)	Inconclusive n (%)	Yes n (%)
0–3 weeks n = 112 (21.4)		**20** **(3.8)**	*92* *(17.6)*	*0*	**108** **(20.7)**	*4* *(0.8)*	*0*	**108** **(20.7)**	*0*	*4* *(0.8)*
4–12 weeks n = 0232 (44.4)		*2* *(0.4)*	**116** **(22.2)**	*114* *(21.8)*	*103* *(19.7)*	**1** **(0.2)**	*128* *(24.5)*	*108* *(20.7)*	**0**	*124* *(23.7)*
3 ≥ 6 months n = 179 (34.2)		*2* *(0.4)*	*1* *(0.2)*	**176** **(33.7)**	*0*	*0*	**179** **(34.2)**	*4* *(0.8)*	*0*	**175** **(33.5)**
Total agreement (%)		**n = 323** **(59.7)**			**n = 288** **(55.1)**			**n = 283** **(54.1)**		

**Table 5:** Step 3: Agreement in 3 × 3 tables between the variables physiotherapist’s intuitive synthesis “risk for functional recovery” and patient-reported variables “pain intensity”, “disability”, “coping”, and “fear avoidance” (n = 523).

	Reported Variables	Pain Intensity (VAS) n = 523			Disability (NDI-DV) n = 523			Active Coping (PCI-A) n = 523			Fear Avoidance (FABQ-A) n = 523		
Intuitive Synthesis		Mild n (%)	Moderate n (%)	Severe n (%)	Mild n (%)	Moderate n (%)	Severe n (%)	Low Risk n (%)	Incon. Risk n (%)	High Risk n (%)	Low Risk n (%)	Incon. Risk n (%)	High Risk n (%)
Low risk n = 43 (8.2%)		**7** **(1.3)**	*29* *(5.5)*	*7* *(1.3)*	**4** **(0.8)**	*26* *(5.0)*	*13* *(2.5)*	**4** **(0.8)**	*23* *(4.4)*	*16* *(3.1)*	**26** **(5.0)**	*7* *(1.3)*	*10* *(1.9)*
Inconcl. risk n = 329 (62.9%)		*3* *(0.6)*	**203** **(38.8)**	*123* *(23.5)*	*2* *(0.4)*	**71** **(13.6)**	*256* *(48.9)*	*56* *(10.7)*	**241** **(46.1)**	*32* *(6.1)*	*19* *(3.6)*	**98** **(18.7)**	*212* *(40.5)*
HigrikDelayed n = 144 (27.5%)		*5* *(1.0)*	*34* *(6.5)*	**112** **(21.4)**	*3* *(0.6)*	*11* *(2.1)*	**137** **(26.2)**	*67* *(12.8)*	*74* *(14.1)*	**10** **(1.9)**	*11* *(2.1)*	*16* *(3.1)*	**124** **(23.7)**
Total agreement (%)		**322** **(61.5)**			**212** **(40.5)**			**255** **(48.8)**			**248** **(47.4)**		

Legend: VAS: Visual Analogue Scale for pain (scores: see Table 1); NDI-DV: Neck Disability Index Dutch Version (scores: see Table 1); PCI-A: Pain Coping Inventory Active (scores: see Table 1); FABQ-A: Fear Avoidance Beliefs Questionnaire Activities (scores: see Table 1).

## Data Availability

Data on patients with WAD were collected and archived in the form of a pen and paper patient record.
